# Extracellular Vesicles Drive Invasive Characteristics and Matrix Remodelling in Organotypic Models of Colorectal Cancer Progression

**DOI:** 10.1002/jex2.70187

**Published:** 2026-09-17

**Authors:** Sonia Guarnerio, Laura Cole, Rawan Maani, Rob Tempest, Alex Mirnezami, Paul Hughes, Stuart Hunt, Christine Le Maitre, Keith Chapple, Nick Peake

**Affiliations:** ^1^ Biomolecular Sciences Research Centre Sheffield Hallam University Sheffield UK; ^2^ NanoFCM Nottingham UK; ^3^ Institute of Developmental Sciences University of Southampton Southampton UK; ^4^ Department of Engineering Durham University Durham UK; ^5^ School of Clinical Dentistry The University of Sheffield Sheffield UK; ^6^ School of Medicine and Population Health The University of Sheffield Sheffield UK; ^7^ Colorectal Surgical Unit Sheffield Teaching Hospitals NHS Foundation Trust Sheffield UK

## Abstract

Colorectal cancer (CRC) remains a leading cause of cancer‐related mortality, with metastasis accounting for over 90% of CRC deaths. While the role of extracellular vesicles (EVs) in cancer progression is recognised, their impact on invasive behaviour and extracellular matrix (ECM) remodelling within physiologically relevant three‐dimensional (3D) microenvironments remains poorly understood. This study utilised quantitative organotypic 3D models to investigate how EVs derived from primary (SW480) and metastatic (SW620) CRC cell lines influence invasion, stromal activation, and ECM remodelling.

We developed models incorporating CRC cells, fibroblasts, endothelial cells, and macrophages to mimic the tumour microenvironment (TME) and lung stroma. EVs isolated from SW480 and SW620 cells were isolated and characterized according to MISEV guidelines and introduced into the models. Treatment with metastatic SW620 EVs significantly enhanced depth and extent of CRC cell invasion compared to primary SW480 EVs or controls, and increased invasion of multicellular clusters. Immunofluorescence analysis revealed elevated expression of cadherin 2 (CADH2) and catenin delta 1 (CTNND1) in SW620 EV‐treated models, indicating involvement of epithelial‐mesenchymal transition (EMT).

In lung stroma models, SW620 EVs reduced matrix stiffness, implying ECM remodelling. Mass spectrometry and multivariate analysis identified distinct proteomic signatures in SW620 EV‐treated models, with significant alterations in collagen type XI expression and unique mass‐to‐charge (m/z) peaks, indicating selective ECM remodelling. SW620 EVs also induced activation of stromal fibroblasts and endothelial cells, as evidenced by increased α‐smooth muscle actin (α‐SMA) and von Willebrand Factor (vWF) expression.

These findings demonstrated that metastatic CRC‐derived EVs enhance invasive behaviour and remodel the ECM, creating a permissive microenvironment for metastasis. The differential effects of primary versus metastatic EVs underscore the importance of tumour stage‐specific vesicle signatures in CRC progression. This study provides a robust 3D model framework to quantify EV‐mediated mechanisms and identify therapeutic targets to disrupt pro‐metastatic communication in CRC.

**Abbreviations**
ANOVA: one‐way analysis of variance
BSA: bovine serum albumin
CHCA: α‐cyano‐4‐hydroxycinnamic acid
CI: confidence interval
CRC: colorectal cancer
DELFIA: dissociation‐enhanced lanthanide fluorescence immunoassay
DMEM: Dulbecco's Modified Eagle Medium
ECACC: European Collection of Authenticated Cell Cultures
ECM: extracellular matrix
EGF: Epidermal Growth Factor
EMT: epithelial‐mesenchymal transition
FBS: foetal bovine serum
H&E: haemotoxylin & eosin
HIAA: heat‐induced antigen retrieval
HPLC: high‐performance liquid chromatography
IMS: industrial methylated spirit
MALDI‐MS: matrix‐assisted laser desorption ionisation mass spectrometry
PCA: Principal component analysis
PLS‐DA: Partial least squares discriminant analysis
PMA: phorbol 12‐myristate 13‐acetate
PS: penicillin/streptomycin
ROI: region of interest
SEC: size‐exclusion chromatography
SEM: standard error of the mean
SMA: smooth muscle actin
TEM: Transmission electron microscopy
TME: tumour microenvironment
VWF: Von Willebrand factor

## 1 Introduction

As the third most common cancer diagnosis and second most common cause of cancer related morbidity, colorectal cancer (CRC) is one of the most prevalent and deadly cancers worldwide (Chen et al. 2025, Wu et al. 2025). Despite efforts to improve screening uptake and methodologies, almost 50% of CRC cases are still only diagnosed in later stages where prognosis is poor (Krilaviciute et al. 2017). In stage III CRC, the cancer has become invasive into the bowel wall, local tissues and lymph nodes, and in stage IV the cancer has become metastatic, spreading to distant organs—frequently the liver or lung (Fadlallah et al. 2024; Shin et al. 2023). Stage IV (metastatic) CRC has a 5‐year survival rate of <10% and around 90% of deaths from CRC are caused by metastasis (Ilyas et al. 2021; Shin et al. 2023), which underscores the need to understand the events leading to disease progression, invasive behaviour and metastatic establishment.

Acquisition of invasive capability is a defining event in the progression towards metastasis. Invasion into local tissues involves breach of the basement membrane, and metastasis to distant organs requires the ability to intravasate into the lymphatic or circulatory system and extravasation into distant metastatic sites (Li et al. 2022; Shin et al. 2023). This progression and these processes require a combination of cellular plasticity and tissue remodelling, driven by biochemical and biomechanical signalling (Nieto et al. 2016; Tape et al. 2024). Successful invasion depends not only on intrinsic changes within tumour cells but also on alterations in the composition and organization of the extracellular matrix (ECM), which facilitate cell migration and dissemination (Friedl and Alexander 2011; Lu et al. 2012; Nieto et al. 2016; Tape et al. 2024).

Cancer cell invasion had previously been thought to primarily occur through single‐cell migration, involving epithelial‐to‐mesenchymal transition to overcome the cellular attachments and tissue organisation maintaining the bowel epithelia (Son and Moon 2010). However, it is becoming clear that there are diverse alternative invasion modalities involving clusters or collective migration of tumour cells, observed in CRC and other cancer types (Friedl and Alexander 2011, Friedl et al. 2012, Lintz et al. 2017; Son and Moon 2010). Such collective invasion has been associated with enhanced metastatic efficiency and resistance to therapy (Friedl and Alexander 2011).

Extracellular vesicles (EVs) have emerged as key mediators of intercellular communication in cancer. Lipid‐bound particles secreted by both cancer and stromal cells, EVs carry bioactive cargo—including proteins, lipids and nucleic acids—that can influence target cells locally and systemically (Lafitte et al. 2019). Through paracrine and endocrine mechanisms, EVs contribute to multiple steps of cancer progression, including invasion, angiogenesis, immune evasion and metastasis (Brown et al. 2024; Johnson et al. 2024; Li et al. 2017). Increasing evidence indicates that cancer‐derived EVs promote epithelial to mesenchymal transition (EMT) and enhance invasive behaviour (Pucci et al. 2023).

EVs have the capability to contribute to both single‐cell and collective invasion behaviour by reprogramming not only cancer cells but also stromal components such as fibroblasts, endothelial cells and macrophages (Pucci et al. 2023; Li et al. 2022; Lafitte et al. 2019). Through these interactions, EVs help establish a permissive microenvironment that supports organotropic invasion and pre‐metastatic niche (PMN) formation in mechanisms shown to involve surface proteins such as integrins and cargo including miRNA (Costa‐Silva et al. 2015; Hoshino et al. 2015; Lafitte et al. 2019; Liu et al. 2020, Si et al. 2024).

While pro‐invasive properties of EVs such as proteolytic enzyme activity, activation of pro‐invasive stromal cell cross‐talk, delivery of pro‐EMT cargo and ECM interaction have been well documented in 2D culture systems (Giusti et al. 2013; Luoto et al. 2023; Raimondo et al. 2015; Rigogliuso et al. 2010; Sun et al. 2018, Xu et al. 2024), these simplified models lack the spatial, mechanical, and cellular complexity of the native tumour microenvironment (TME). EV biogenesis and cargo composition differ between monolayer and 3D cultures, leading to changes in vesicle function (Christianson et al. 2013, Franzén et al. 2015), and the biomechanical properties associated with the desmoplastic, fibrotic tissue microenvironment in CRC also influence EV properties (Powsner et al. 2025, Xu et al. 2025). Thus, understanding how EVs contribute to invasive behaviour requires experimental models that capture the three‐dimensional organization, stromal interactions and dysfunctional tissue microenvironment characteristic of human tumours.

We have previously utilised organotypic 3D models to understand CRC growth and invasion within a complex and dynamic TME (Delaine‐Smith et al. 2019). By incorporating multiple cell types, such as fibroblasts, endothelial cells and immune cells within and collagen‐rich ECM, they recapitulate these interacting features of the TME and allow direct visualisation of invasive behaviour. Our previous work has demonstrated that in CRC models, co‐cultures with fibroblasts promote spontaneous spheroid formation and invasion and drive ECM cross‐linking and stiffening (Delaine‐Smith et al. 2019). Further, organotypic models of squamous carcinoma models (Nyström et al. 2005) have enabled quantitative assessment of invasion dynamics, making these systems well suited for studying EV‐mediated modulation of the ECM and cancer cell behaviour.

The present study aimed to adapt and utilise quantitative organotypic 3D models of CRC progression to investigate how cancer‐derived EVs influence invasion and ECM remodelling. By comparing the responses to EVs derived from primary (SW480) and metastatic (SW620) CRC cell lines, we sought to determine how EV origin and tumour stage affect invasive capacity, stromal cell interaction and activation, and matrix properties. By using two distinct modelling approaches, we also aimed to unpick the effect of EVs directly on the invasive behaviour of CRC cells using an organotypic collagen model exposed to exogenous EVs, before then utilising a model based on lung stromal physiology to mimic EV conditioning of a metastatic site—applying EVs and analysing microenvironmental alterations prior to assessing subsequent CRC invasion. This approach provides a physiologically relevant framework to understand how EVs contribute to the transition from local invasion to metastatic dissemination in CRC.

## 2 Materials and Methods

### 2.1 Cell Culture and Maintenance

Primary adenocarcinoma SW480 cell line, secondary adenocarcinoma SW620 cell line, human leukaemia monocytic cell line THP‐1 and microvascular endothelial cell line HULEC‐5a were purchased from the European Collection of Authenticated Cell Cultures (ECACC). Human lung fibroblast cell line (MRC5) was purchased from Sigma Aldrich (Gillingham, UK). SW480, SW620 and MRC5 cells were cultured in complete Dulbecco's Modified Eagle Medium (cDMEM), which contained high glucose (4.5 g/L) DMEM, GlutaMAX, pyruvate (Gibco, Thermo‐Fisher, UK), 10% v/v foetal bovine serum (FBS, Gibco, Thermo‐Fisher, UK) and 1% v/v penicillin/streptomycin (PS, Lonza Ltd., UK). THP‐1 cells were cultured in RPMI 1640 basal media (Gibco, Thermo‐Fisher, UK) with 10% v/v FBS and 1% v/v PS. HULEC‐5a cells were cultured in basal MCDB131 (Gibco, Thermo‐Fisher, UK), supplemented with 10 ng/ml Epidermal Growth Factor (EGF; Fisher Scientific; Loughborough, UK), 1 µg/ml hydrocortisone (Merck, UK), 10 mM Glutamine (Gibco, Thermo‐Fisher, UK), 10% v/v FBS and 1% v/v P/S. All cells were maintained in a humidified incubator at 37°C and 5% CO_2_ in air. Culture medium was replaced every 3–4 days and cells sub‐cultured (ratios 1:10 SW480/SW620, 1:5 MRC5/THP‐1/HULEC‐5a) with Trypsin‐EDTA (Thermo‐Fisher, Loughborough, UK) when reaching 70%–80% of confluence. Cells were checked for mycoplasma with MycoAlert Detection kit (Lonza Ltd., UK) every 6 months and were shown to be negative throughout.

### 2.2 Isolation of CRC EVs

SW480 and SW620 EVs were isolated as previously described (Guarnerio et al. 2023). Briefly, SW480 or SW620 cells were cultured in WHEATON CELLine AD‐1000 Bioreactor flasks (DWK Life Sciences, GmbH) in 10% v/v Gibco EV‐depleted FBS (Thermo‐Fisher, UK) and DMEM, to avoid serum EV contamination. EV‐enriched conditioning media (CM) was harvested weekly, alongside media replacement. Conditioning media was centrifuged at 300 × g for 5 min, supernatant centrifuged again at 2000 × g for 5 min, then concentrated to reach a volume of 0.5 mL and ultrafiltered through Vivaspin 20 (100 kDa MWCO) (Sartorius, Germany) at 3000 g. EVs were separated from soluble factors by size‐exclusion chromatography (SEC) loaded in Econo‐Pac columns (Biorad, Watford, UK) with 10 mL sepharose CL‐2B (GE Healthcare, Uppsala, Sweden) and eluted in PBS in 500 µL fractions.

### 2.3 EV Quantification

Bicinchoninic acid (BCA) assay was performed according to manufacturers instructions (Thermo Fisher, USA). EV size and particle count was assessed using a nanoanalyser instrument based on nano‐flow cytometry using the nanoanalyser (NanoFCM, MediCity, Nottingham, UK). EVs were compared to QC beads (250 nm silica standard) and size beads (68, 91, 113 and 155). Relative levels of tetraspanin expression were assessed using dissociation‐enhanced lanthanide fluorescence immunoassay (DELFIA, Revvity, UK), as previously described (Guarnerio et al. 2023), using antibodies to CD9 (ab2215 mouse monoclonal [MEM‐6]; Abcam, UK; 1:5000 dilution), CD63 (MCA2142 mouse monoclonal [MEM‐259]; Bio‐Rad Ltd, UK; 1:5000 dilution) and CD81 (MCA1847 mouse monoclonal [1D6]; Bio‐Rad Ltd, UK; 1:5000 dilution).

### 2.4 Western Blotting for TSG101 and GM130

To confirm the presence of EVs, positive (TSG101) and negative (GM130) markers of EVs were investigated by western blotting. Cell lysates were used as negative controls to evaluate marker specificity. Antibodies against TSG101 (ab2386 mouse monoclonal [CUB 7402]; Abcam, UK; 1:1000 dilution) and Golgi matrix protein GM130 (ab52649 rabbit monoclonal [EP892Y]; Abcam, UK; 1:1000 dilution) were used to probe 15 µL of EV preparations following separation by SDS‐PAGE and transfer to nitrocellulose membrane using a Trans‐Blot Turbo transfer system (Bio‐Rad, Watford, UK) for 7 min, and visualised using the Li‐Cor Odyssey instrument (LI‐COR Biosciences, Cambridge, UK).

### 2.5 Transmission Electron Microscopy (TEM)

TEM of EVs was performed at the Electron Microscopy facility, Faculty of Science, University of Sheffield. EVs were transferred through absorption onto carbon‐coated copper grids for 1 min, quickly dried with filter paper and then rinsed twice with water. EVs were stained for 2 min in filtered 2% uranyl acetate. The grid was allowed to dry for 10 min before staining in uranyl formate. Grids were visualised on a FEI Tecani G2 Spirit BioTwin (PennState, USA) TEM, and images were recorded using a Gatan Orius 1000B CCD camera and Gatan Digital Micrograph software (Gatan, USA).

### 2.6 Organotypic Invasion 3D Model

A basal invasion 3D model was composed of 57.5% v/v collagen type I (3 mg/ml, BD Bioscience, Wokingham, UK) and 17.5% v/v Geltrex, which is a mixture of basement membrane components (Life Technologies, Thermo‐Fisher, UK). The mixture was prepared in cDMEM to which MRC5 fibroblasts were mixed at a concentration of 528,000 cells/ml. Gels were cast using 125 µL/well in the upper compartment of a 24‐well Thincert cell culture insert with 0.4 µm pore size (Greiner Bio‐One, Stonehouse, UK) for 1 h at 37°C and 5% CO_2_. Then, 100 µL of SW480 at 264,000 cells/ml in serum‐free DMEM were added to the top of the gel. Complete DMEM (600 µL) was added in the compartment underneath as a chemoattractant for the cancer cells.

### 2.7 Lung Stroma 3D Model

A 3D model of lung stroma was developed by adding MRC5 (2,112,000 cells/ml), THP‐1 (700,000 cells/ml) and HULEC‐5a (1,200,000 cells/ml) in the mixture of collagen type I/Geltrex to recreate lung stromal tissue. THP‐1 monocytes were differentiated in M0 macrophages (dTHP‐1) 72 h before the 3D culture with 50 ng/ml of phorbol 12‐myristate 13‐acetate (PMA, ThermoFisher, UK). To monitor the cell ratio, cells were stained for 30 min with fluorescent probes before the inclusion in the gel; for MRC5, 10 µM of CellTracker green CMFDA (ThermoFisher, UK), for HULEC‐5a, 10 µM of CellTracker Red CMTPX (ThermoFisher, UK) and 500 nM of CellTracker Deep Red dye (ThermoFisher, UK) for differentiated THP‐1 in serum free culture media were used. Ultimately, the three cell lines were added to the gel mixture at appropriate experimental density and ratio. The gel was left to set for 1 h at 37°C and 5% CO_2_, and then cultured 7 or 14 days in cDMEM supplemented with 0.05 mM ascorbic acid 2‐phosphate (Sigma). The growth media was changed every 3–4 days.

### 2.8 Conditioning With CRC EVs

Routine treatments with EVs were applied normalised to protein concentration. To observe EV uptake, SW480 and SW620 EVs were labelled with 500 nM CellTracker Deep Red (Thermo‐Fisher, UK) for 30 min and then ultracentrifuged for 1 h at 100,000 g with an Optima MAX Ultracentrifuge (Beckman‐Coulter, High Wycombe, UK) to remove the excess of dye, then 50 µg/ml of SW480 EVs or SW620 EVs were introduced in serum free DMEM in the upper compartment of the transwell models. After EV conditioning, lung 3D models were removed from transwells and placed in ultra‐low attachment 96‐well plates (illustrated in Supplementary Figure 1). SW620 were labelled with 10 µM Red CMTPX dye for 30 min and then introduced in the 3D model at a concentration of 264,000 cells/ml. The cells were left to invade the gel for 3 days before formalin fixation and analysis.

### 2.9 Processing and Analysis of 3D Models

3D models were processed at different end points for paraffin embedding according to the standard protocols. Briefly, samples were fixed overnight in 10% neutral buffered formalin (Merck, UK), then dehydrated in graded industrial methylated spirit (IMS; Merck, UK), cleared in xylene substitute (Sub‐X, Leica Microsystems, UK) and embedded in molten paraffin wax (Leica Microsystems, UK). Tissue sections (4 µm) were obtained with a microtome and mounted onto X‐tra adhesive glass slides (Leica Microsystems, UK) and dried for a minimum overnight and for a maximum of a week at 37°C. The sections were stored at room temperature (RT) until further analysis. Prior to any staining, deparaffinisation was performed by washing in Sub‐X (3 × 5 min). Then, sections were rehydrated in graded passages of IMS (100% x 2 min, 90% x 2 min, 70% x 2 min) and rinsed in water. Histological and immunohistochemical images were observed with an Olympus BX60 microscope and images captured by a digital camera Olympus XC30 and Olympus CellSens software (Media Cybernetics, Buckinghamshire, UK). Mechanical testing was performed using an Ametek CS2 5 kN force measurement tester, with a CLC250 load cell.

### 2.10 Haematoxylin and Eosin Staining

Sections were dyed with Mayer's Haematoxylin (Merck, UK) for approximately 3 min, blued in running tap water for 6 min before the staining for 4 min with 1% aqueous Eosin Y solution (Leica biosystems, UK). Sections were dehydrated in IMS (5 min, 3 times), and then IMS was cleared by Sub‐X (5 min, 3 times). Finally, sections were mounted with 1 drop of Pertex (Leica Biosystems, UK) per slide and coverslips applied.

### 2.11 Masson Trichrome Staining

Masson Trichrome staining was applied to the sections according to the manufacturers protocol (Atom Scientific, UK). Briefly, nuclei were stained with Weigert's Iron haematoxylin for 20 min, rinsed in 1% acid alcohol solution and blued in tap water. Ponceau fuchsin was applied for 5 min and differentiated in phosphotungstic acid for 15 min. Then, the samples were transferred without rinsing into methyl blue solution for 5 min. Sections were finally rinsed, dehydrated in IMS and cleared by Sub‐X. Finally, sections were mounted with 1 drop of Pertex (Leica Biosystems, UK) per slide and coverslips applied.

### 2.12 Immunohistochemistry

After deparaffinisation and rehydration, endogenous peroxidases were blocked by submerging the slides in 3% v/v hydrogen peroxide and 0.75% HCl (Merck, UK) in IMS, then underwent either heat‐induced antigen retrieval (in 10 mM citric acid, pH 5.9) or enzyme antigen retrieval (using 0.01% α‐chymotrypsin). Secondary antibody host interactions were blocked with a solution containing 1% w/v BSA and 25% v/v normal goat serum (Merck, UK), before incubation with primary antibodies in 1% BSA overnight (Ki67, ab15580, 1:200; α‐SMA, ab7817, 1:1000; CD68, ab213363, 1:8000; vWF, ab9378, 1:300). Visualisation was undertaken after incubation with appropriate secondary antibody for 30 min at RT, then horseradish peroxidase avidin‐biotin complex (HRP ABC kit, Vector Laboratories, Peterborough, UK) for 30 min at RT, and finally 0.65 mg/ml of 3,3‐diaminobenzidine tetrahydrochloride (Merck, UK). Slides were washed in dH2O for 5 min prior to the nuclei counterstain in Mayer's haematoxylin (approximately 3 min) and then blued under tap water for 6 min.

### 2.13 Immunofluorescence

To evaluate ECM‐stroma interactions, immunofluorescence (IF) was performed using primary antibodies to cadherin 2 (CADH 2, N‐cadherin CD235, Thermo Fisher, UK, 1:50), cadherin 1 (CADH1, E‐cadherin ab40772, Abcam 1:100) and catenin delta‐1 (CTNND1, p120 ab92514, Abcam 1:100). After deparaffinisation and rehydration, heat‐induced antigen retrieval was performed followed by blocking in goat serum. For p120, a permeabilisation step in 0.1% Triton‐X/TBS for 10 min was also undertaken. Sections were incubated with primary antibodies overnight in 1% BSA at 4°C in a humidified environment protected from light. Proteins were visualised following incubation with secondary antibodies in 1% w/v BSA for 1 hr at RT before mounting in VECTASHIELD HardSet Antifade Mounting Medium with 4′,6‐diamidino‐2‐phenylindole (DAPI) (Vector Laboratories, Peterborough, UK). Images were acquired with Olympus IX81 inverted microscope (Media Cybernetics, Buckinghamshire, UK). Confocal images were acquired with Zeiss Axio LSM800 (Zeiss, Germany, UK).

### 2.14 Proteomic Analysis of 3D Models

Enzyme‐linked immunosorbent assay (ELISA) was performed on culture media of the 3D lung models according to manufacturer's instructions (IL‐6, IL‐8, MCP‐1 from PeproTech EC Ltd., UK; TIMP‐1, TIMP‐2 from Bio‐Techne, UK). For matrix‐assisted laser desorption ionisation mass spectrometry (MALDI‐MS) analysis, paraffin was removed from the sections by incubating for 20 min at 60°C, followed by washes in high‐performance liquid chromatography (HPLC) grade toluene (Merck, UK) (3 × 4 min). Sections were rehydrated through washes in 100%, 90% and 70% (3 min each) of HPLC‐grade ethanol and then rinsed in de‐ionised water (2 × 3 min). Heat‐induced antigen retrieval was performed using 10 mM Citric Acid at 97°C in a water bath for 30 min. Sections were then left to stand for 5 min within the hot buffer and placed in dH2O for 5 min. MS‐grade Trypsin Gold (Promega, UK) at 20 µg/mL was spotted (1–5 µL). Sections were then left in a humidity chamber overnight at 39°C.

### 2.15 Mass Spectrometry Profiling

α‐cyano‐4‐hydroxycinnamic acid (CHCA) matrix was spotted (0.8 µL) onto the whole sample at a concentration of 5 mg/mL in a 70:30 acetonitrile:H2O with 0.4% v/v of trifluoroacetic acid (TFA). Analysis were performed using a MALDI Select Series MRT (Waters Corporation, Manchester, UK) quadrupole TOF (qTOF). External calibration was performed by using phosphorus red (Merck, UK). MALDI‐MS profiling was acquired in positive ion mode at a range of m/z 0 to 2400. Mass spectra were visualised with mMass open‐source software. All mass spectrometry spectra are stored at the Sheffield Hallam University Research Archive (SHURA, https://shura.shu.ac.uk/) and can be made available on request to the corresponding author.

### 2.16 Multivariate Analysis

Average spectra were exported as *.txt files, transformed into .csv and uploaded in MetaboAnalyst. Then, statistical analysis (one factor) was selected and peaks were aligned through pre‐processing with mass tolerance of 0.025. Data filtering was applied with interquartile range (IQR) filters to discard variables that were unlikely to be of use. Finally, normalisation was obtained by sum and auto‐scaling. Principal component analysis (PCA), Partial least squares discriminant analysis (PLS‐DA) and hierarchical clustering were automatically performed.

### 2.17 Statistical Analysis

All statistical analysis was performed using Prism 8.1.1. Data were tested for normality (gaussian distribution) with two modalities: D'Agostino & Pearson test and Shapiro‐Wilk test. Multiple comparison through ordinary one‐way analysis of variance (ANOVA) with Tukey's test as post‐hoc analysis was performed for parametric data, while Kruskal–Wallis test with Dunn's test as post‐hoc analysis was performed for non‐parametric data. *P*‐values <0.05 were considered significant.

Data representation varied according to the number of replicates and the normal distribution. For data with n ≤ 6, individual values were shown. When data were shown as summary, mean ± standard error of the mean (SEM) was used for parametric data, whereas median ± 95% confidence interval (CI) was used for non‐parametric data.

## 3 Results

### 3.1 Isolation and Characterisation of SW480 and SW620 CRC EVs

**FIGURE 1:**
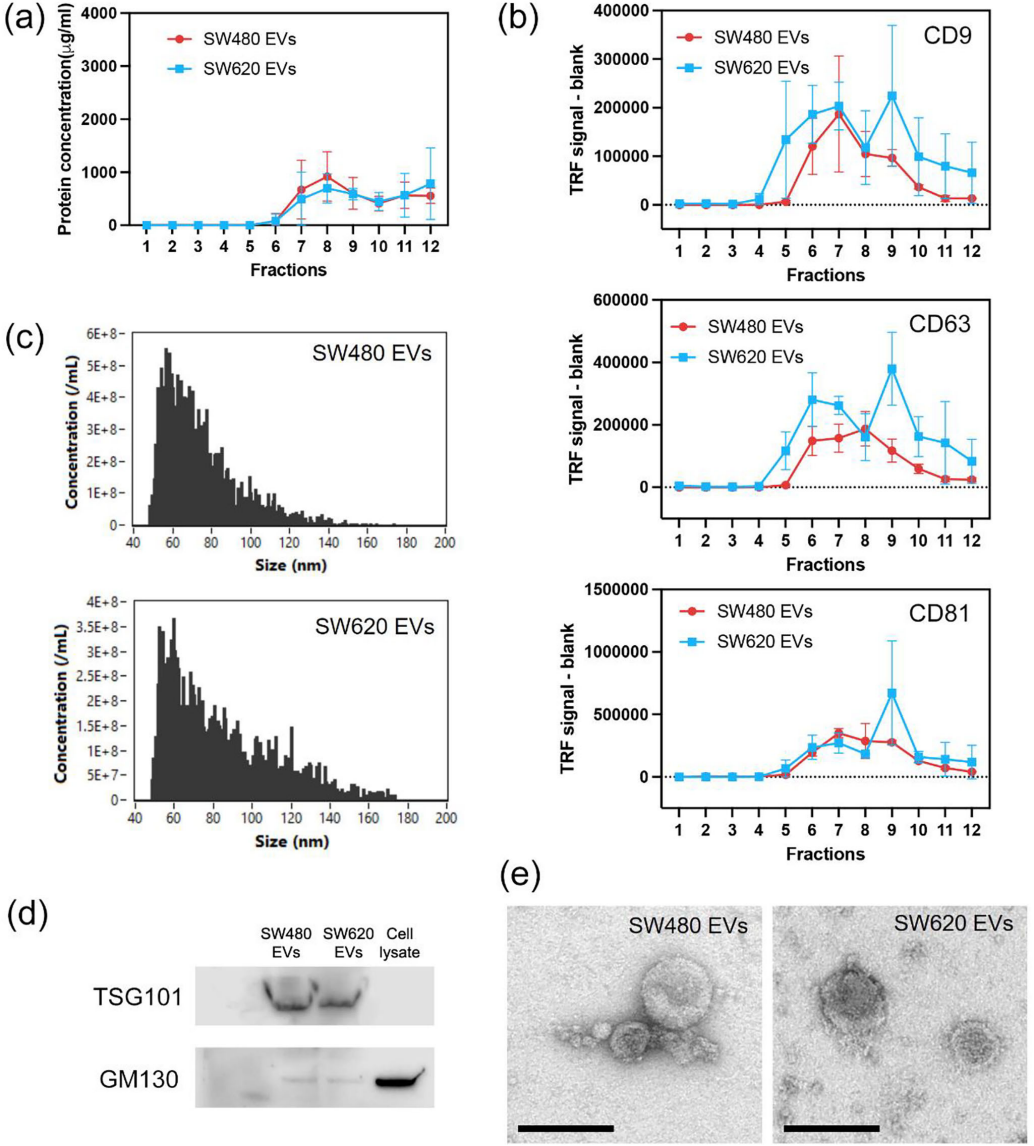
SW480 and SW620 EV characterisation. (a) BCA assay of 1–12 SEC fractions collected from SW480 and SW620 cell lines. (b) DELFIA‐ELISA assay for tetraspanin CD9, CD63 and CD81 on 1–12 SEC fractions from SW480 and SW620 cell lines. (c) Nanoflow cytometry histograms showing size and concentration of SW480 and SW620 EVs. (d) Western blot for TSG101 EV marker and GM130 Golgi marker on pooled SW480/SW620 EVs compared to cell lysate. (e) SW480 and SW620 EVs morphology with TEM. Scale bar = 100 nm.

EVs from CRC cell lines SW480 and SW620 were obtained through SEC and characterised according to MISEV guidelines (Welsh et al. 2024). Characterisation showed comparable results to previous analysis performed by our group (Guarnerio et al. 2023). High protein concentration was found in fractions 7‐8‐9, reaching 1000 µg/ml at fraction 7 for both cell lines (Figure 1A). Fractions 6–10 were also positive for tetraspanin CD9, CD63, CD81 markers (Figure 1B). EVs originating from SW620 showed a fluorescence peak at fractions 9 for all the three markers, suggesting a population of smaller EVs positive particularly for CD63 from this line—although variability was high for CD9 and CD81. Fractions 7–9 were pooled and characterised for size and concentration analysis. The EV size distribution assessed by nanoflow cytometry was similar between the two cell lines (mean ± SD 75.7 ± 0.99 for SW480, and 79.8 ± 3.02 for SW620, Figure 1C). Western blot showed the luminal EV marker TSG101 in both SW480 and SW620 EVs, whilst low positivity of anti‐Golgi marker GM130 was observed compared to cell lysate (Figure 1D). Intact morphology and EVs with different sizes were observed with TEM (Figure 1E).

### 3.2 Development of 3D Organotypic Models to Evaluate CRC Invasion and Metastasis

**FIGURE 2:**
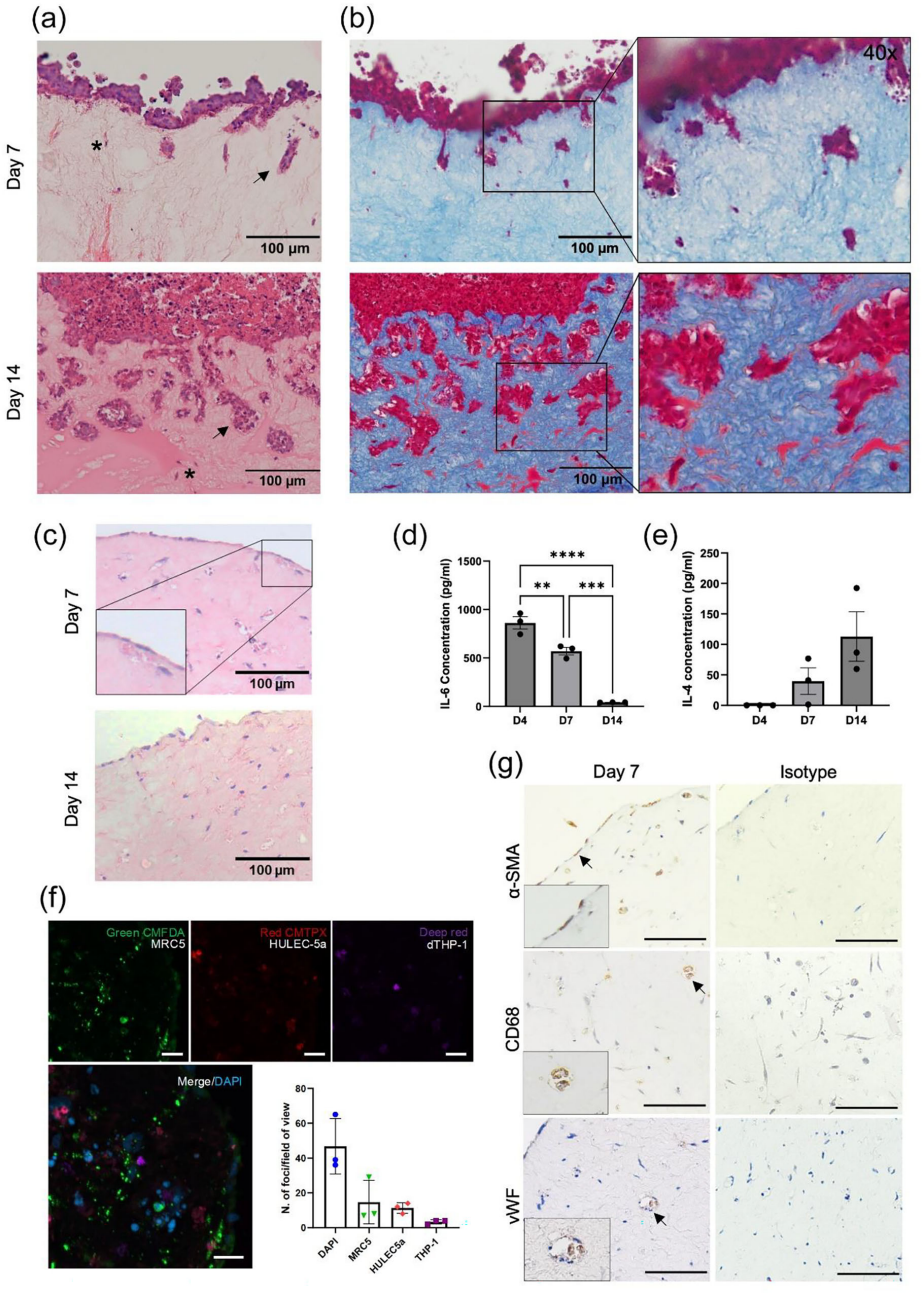
Organotypic 3D model characterisation. (a) Haematoxylin/eosin of day 7 and day 14 culture of the organotypic model of cancer invasion. Asterisks indicate examples of fibroblasts embedded within the matrix, and black arrows indicate examples of invasive clusters following their introduction into the gel. (b) Masson's Trichrome showing collagen fibre alignment. (c) Haematoxylin/eosin of day 7 and day 14 of the organotypic model lung stroma. (d) Pro‐inflammatory IL‐6 secretion at day 4, 7 and 14. Mean ± SEM (*n* = 3). ANOVA, ** *p* < 0.01, *** *p* < 0.001, **** *p* < 0.0001. (e) Anti‐inflammatory IL‐4 secretion at day 4, 7 and 14. Mean ± SEM (*n* = 3). ANOVA. (f) Quantification of cells with passive dyes (*n* = 3). Scale bar = 20 µm. (g) Immunohistochemical staining of α‐SMA, CD68 and vWF at day 7 of culture. Scale bar = 100 µm.

To assess the impact of EVs directly on invasive behaviour, MRC5, a fibroblast cell line derived from tissue of a 14 week old foetus was mixed with collagen type I and Geltrex in the organotypic culture to. To model CRC cell invasion and develop an assay that would enable us to assess the impact of primary (SW480) and metastatic (SW620) EVs on this, SW480 cells were introduced on the top layer of the culture after the gel set and the organotypic models were harvested at day 7 and day 14 of culture for characterisation. Clusters of SW480 were observed at both timepoints and were morphologically discernible from MRC5 fibroblasts. MRC5 showed an elongated shape and were found as single cells dispersed in the collagen matrix (Figure 2A). At day 14, SW480 cell clusters appeared bigger in size and more abundant. The rich collagen‐based matrix was visible with Masson's Trichrome stain in blue and showed cross‐linked fibrillar structures of collagen (Figure 2B).

After establishing this organotypic invasion model, we then developed a second system that enabled EV conditioning—assessing the formation of a permissive lung stromal microenvironment by CRC EVs and subsequent impacts on CRC cell invasion. HULEC‐5a and dTHP‐1 were then mixed with MRC5 in the gel to mimic the stroma of the lung and cultured for 7 and 14 days. The ratio chosen between the three cell lines was 52.5:30:17.5 (MRC5:HULEC‐5a:dTHP‐1) based on anatomical realism according to literature (Crapo et al. 1982, Sahai et al. 2020). Morphology was investigated with H&E staining (Figure 2C). At day 7, cells with different morphology can be observed within the gel matrix. Of note, a single layer of tightly closed elongated cells was observed at the border of the culture (Figure 2C).

Secretion of cytokines MCP‐1, IL‐6, IL‐4 and IL‐10 into the culture media were investigated to evaluate the inflammatory condition of the 3D model at day 4, 7 and 14. Levels of the pro‐inflammatory cytokine IL‐6 were significantly lower (*p* < 0.01) at day 7 compared to day 4 (Figure 2D). A complete loss of IL‐6 was observed at day 14, thus significantly decreasing from day 4 (*p* < 0.0001) and day 7 (*p* < 0.001, Figure 2D). A similar trend was observed for MCP‐1, which mediates monocyte recruitment, however the decreases were not significant (Supplementary Figure 2). The anti‐inflammatory cytokine IL‐4 showed an opposite trend, with gradual increase from day 4 to day 14 (non‐significant, *p* = 0.19, Figure 2E). These results suggest a shift in inflammatory profile in the culture. No significant differences were seen for IL‐10 which is also associated with anti‐inflammatory activity (Supplementary Figure 2).

To track the three cell lines introduced in the collagen matrix, cells were incubated with CellTracker fluorescent probes (green for MRC5, red for HULEC‐5a and deep red for dTHP‐1) before the 3D culture and at day 7 were observed under the microscope (Figure 2F, Supplementary Figure 3). Intact cells were identified via DAPI counterstain and classified according to the dyes surrounding the nucleus. Despite the relative proportion in which the cells were introduced in the culture remaining constant, debris and apoptosis was observed by DAPI staining. Moreover, a percentage of intact cells were not positive for any of the dyes, indicating a possible loss of stain during the long culture. Markers associated with the cell types introduced in the lung organotypic culture were further characterised with IHC; α‐SMA for MRC5, CD68 for dTHP‐1 and vWF for HULEC‐5a. Cells positive for α‐SMA were found at day 0, gradually decreasing at day 7 and at day 14 (Figure 2G, Supplementary Figure 4). Cells in the border of the gels were also positive for α‐SMA which is a characteristic stress fibre marker of activated fibroblasts and the most widely used marker of the myofibroblastic cancer‐associated fibroblast (Travaglini et al. 2020). CD68 was identified only in large rounded cells, which is the typical morphology of macrophages (Figure 2G). Lastly, vWF was found in a small number of cells, occasionally creating clusters of two or three cells around pores in the matrix (Figure 2G). Isotype controls were negative for all the three markers.

### 3.3 Metastatic CRC EVs Induce Invasion of Primary Cancer Cells

**FIGURE 3:**
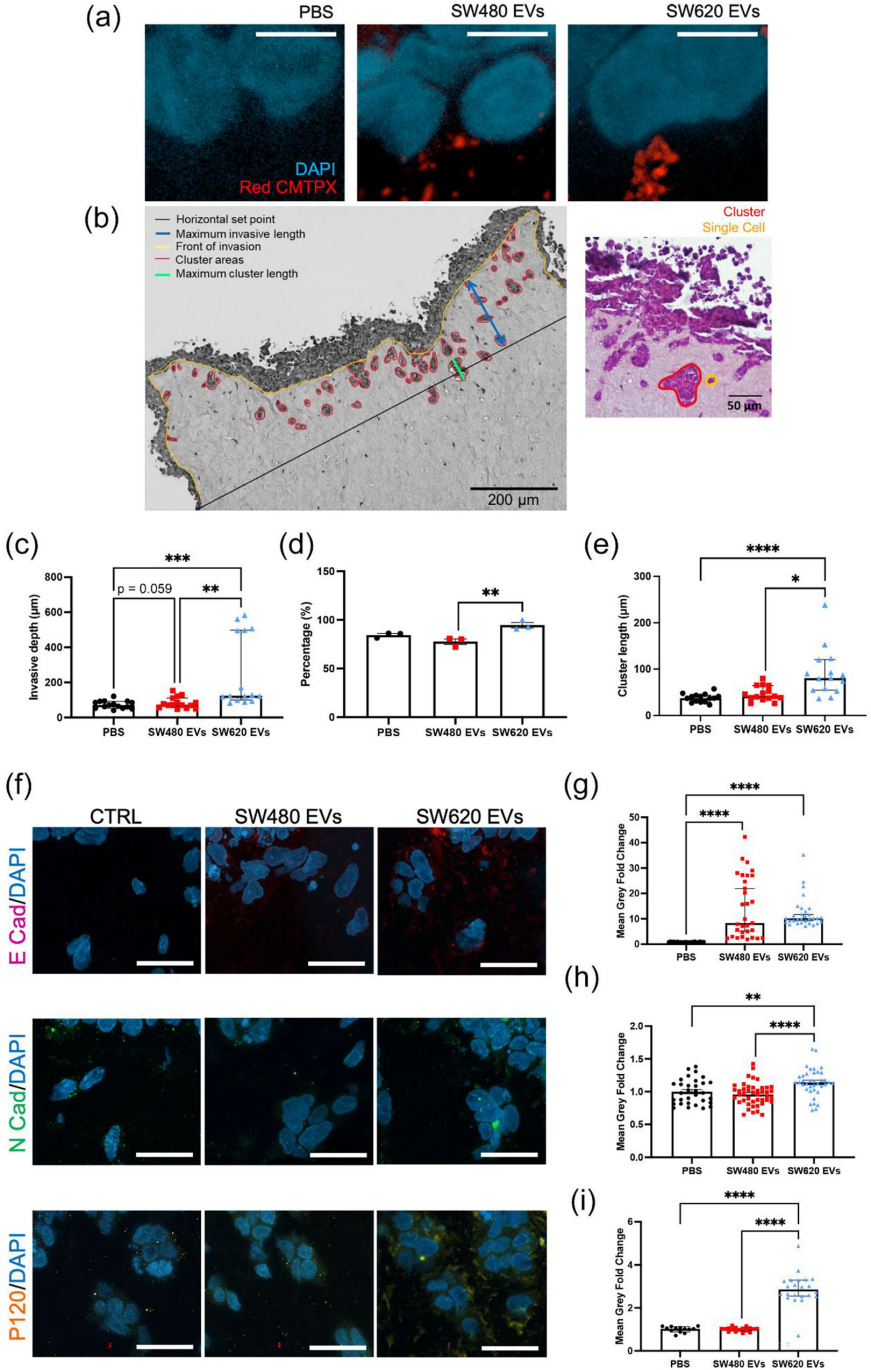
Organotypic model conditioning of CRC invasion with CRC EVs. (a) Representative confocal images of EVs (red) in the organotypic model at day 7 of culture. DAPI counterstain for cell nuclei. Scale bar = 5 µm. (b) Quantitative measurements of CRC invasion. All the analyses were performed with ImageJ 1.5i software. Scale bars from the images were used to set the scale of the measurements. The horizontal set point was taken considering the invasive front of cancer cells always at the top of the sample. The perimeter dividing cancer cells and matrix was defined as front of invasion. Freehand selections were drawn following the area of the SW480 cells or cluster cells invading the matrix and area size were acquired. A threshold of 70 µm2 (which corresponded to a single nuclei observation) was considered when separating between single cells and cell clusters. Five measurements of the length of invasion were taken where the clusters were found at the furthest distances from the invasive front. The five biggest clusters observed in the samples were also measured in their vertical length to see whether EVs impacted on the direction of invasion towards the matrix. (c) Depth of invasion. Measurements were taken in the regions where the invasion was > 50 µm inside the matrix. Median + CI 95% (*n* = 3); Kruskal–Wallis, ** *p* < 0.01, *** *p* < 0.001. (d) Percentage of SW480 clusters on the total amount of invasive foci. Mean ± SEM (*n* = 3). One‐way ANOVA, ** *p* < 0.01. (e) Length of SW480 clusters invading the matrix. Measurements were taken of the 10 biggest clusters/sample that were detached from the invasive layer. Median + CI 95% (*n* = 3); Kruskal–Wallis, * *p* < 0.05, **** *p* < 0.0001. (f). Representative IF images of the invasive layer stained for CADH1 (E‐Cadherin, purple), CADH2 (N‐Cadherin, green) and CTNND1 (P120, yellow). DAPI counterstain for cell nuclei. (g) Mean grey intensity of CADH1 (E‐Cadherin, fold change). Median + CI 95% (*n* = 3); Kruskal–Wallis, **** *p* < 0.0001. (h) Mean grey intensity of CADH2 (N‐Cadherin, fold change). Mean ± SEM (*n* = 3); one‐way ANOVA, ** *p* < 0.01, **** *p* < 0.0001.(i) Mean grey intensity of CTNND1 (P120, fold change). Median + CI 95% (*n* = 3); Kruskal–Wallis, **** *p* < 0.0001.

Firstly, EV retention in the matrix‐rich models was assessed by labelling EVs with CellTracker Deep Red. At day 7 of the culture, fluorescence was observed in both treated gels, whereas no staining was identified in the untreated gels (Figure 3A). To investigate whether EVs had an impact on the invasion profiles of SW480, quantitative measurements were taken based on stained sections from the 3D invasive culture treated with CRC EVs for 7 days (Figure 3B). Depth of invasion, invasive clusters percentage and length were quantified. SW620 EVs significantly increased the depth of invasion compared to control (*p* < 0.001, Figure 3C). Upon treatment with SW620 EVs, an increase of multicellular clusters was observed compared to SW480 EV treatment (*p* < 0.01, Figure 3D) and control (ns, *p* = 0.059). SW480 and SW620 EVs both impacted on cluster length and areas, although only the SW620 EVs increase was significant (*p* < 0.0001, Figure 3E and Supplementary Figure 5). Interestingly, both depth of invasion and cluster length were significantly different when the two EV treatment were compared (depth *p* < 0.01, length *p* < 0.05, Figure 3C,E). Expression of markers of invasion in the invasive layer was investigated through IF. Cadherin 1 (CADH1) expression significantly increased in both SW480/SW620 EV treated cultures compared to the control (*p* < 0.0001, Figure 3F,G). An increase in Cadherin 2 (CDH2) was identified in 3D models treated with SW620 EVs which was statistically significant from the PBS control (Figure 3F, H, *p* < 0.01) and the SW480 EVs condition (*p* < 0.0001). Similarly, catenin delta 1 (CTNND1, p120) increased with SW620 EVs, significantly compared to the PBS control and SW480 EVs treatment (Figure 3F, I, *p* < 0.0001). Alterations to release of inflammatory cytokines from the models was not significantly altered after treatment with EVs (Supplementary Figure 6), suggesting that altered invasion was separate from the formation of an inflammatory niche.

### 3.4 Impact of CRC EVs on Structural Matrix Proteins in the Organotypic Culture of Lung Stroma

**FIGURE 4:**
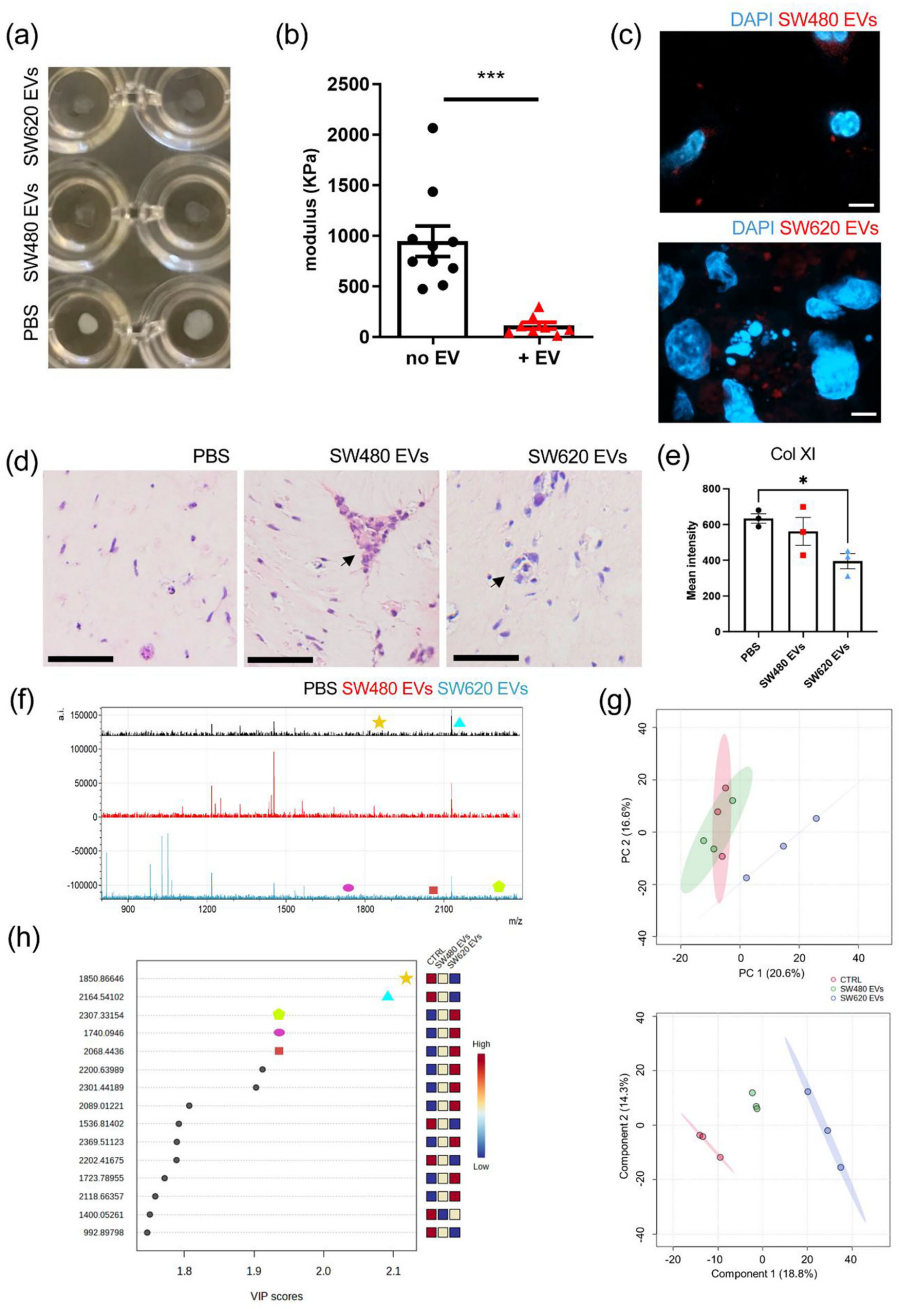
Proteomic analysis of 3D organotypic model of lung stroma after treatment with CRC EVs. (a) Representative photograph of the lung stroma organotypic model in the wells, showing relative opacity of the gels. (b) mechanical testing of lung collagen models after treatment with SW480 EVs. (c) Representative images of SW480 and SW620 EV retention in the gel (red). Cell nuclei counterstained with DAPI. Scale bar = 5 µm. (d) Representative images of H&E staining of the 3D model of lung stroma. Black arrows = cell aggregates. Scale bar = 50 µm. (e) Collagen type XI expression. Mean ± SEM (*n* = 3). One‐way ANOVA, * *p* < 0.05. f) Average mass spectra obtained from the profiling of organotypic culture of lung stroma. Mass range 800–2400 m/z. Control spectrum in positive offset, SW620 EVs spectrum in negative offset, and symbols indicate specific m/z peaks of interest. (g) Unsupervised PCA and supervised PLS‐DA showing how the spectra clustered. CTRL = PBS control. h) VIP score showing the top 15 discriminatory m/z signals between the groups, with symbols indicating peaks linked to the spetra shown in panel e. CTRL = PBS control.

SW480 and SW620 EVs were added to the culture media of the lung from day 1 to day 7 to evaluate whether changes in the PMN could be observed upon cancer EV conditioning. After 7 days of culture, differences in the opacity of the gels were observed (Figure 4A). Both SW480 and SW620 EV treatments reduced the opacity of the gels compared to the control, suggesting a loss of matrix stiffness, which was then corroborated by mechanical testing of the gels which illustrated a significant decrease in modulus after treatment with EVs (Figure 4B, *p* < 0.0001). EV uptake in the collagen matrix was confirmed as labelled EVs were found in the middle of the gel in both conditions (Figure 4C). Gels conditioned with SW480 and SW620 EVs contained cell aggregates, which were not found in the control (Figure 4D).

To understand whether the morphological changes observed could also impact the model at a molecular level, cell and matrix protein markers were evaluated. Decrease of expression of α‐SMA was observed with SW620 EVs, while CD68 expression was not affected by the EV conditioning (Supplementary Figure 7). Non‐significant increase of vWF was observed in cultures treated with both CRC EVs, which was higher following SW620 EV stimulation (*p* = 0.1152) (Supplementary Figure 7). Collagen III, collagen VI and collagen XI, which have been associated with cancer progression, were screened through dot blots (Figure 4E, Supplementary Figure 8). Despite the low intensity, collagen XI decreased after SW620 EV treatment (*p* < 0.05, Figure 4E). Decrease in collagen XI was also observed after SW480 EV treatment, but this was not statistically significant (figure 4E).

Mass spectrometry was used as a screening method to assess changes induced in the 3D models by EV treatments. Clear differences in the average spectra profile were observed (Figure 4F). Specifically, SW620 EV treatment showed intense signals between 900 and 1100 m/z which were absent in both control and treatment with SW480 EVs. Multivariate analysis was then performed to evaluate the significance of the changes and to putatively attribute the differences to specific m/z signals (Figure 4G‐H). Unsupervised PCA analysis confirmed that the 3D models treated with SW620 EVs showed the major differences, as they separated from the other two groups (Figure 4G). By applying a supervised approach via PLS‐DA, a separation between all the three groups was observed and each group clustered independently from the others. Variable importance in projection (VIP) scores were obtained from PLS‐DA (Figure 4H). Signals that distinguished between the three groups of samples ranged from 992 to 2369 m/z. The peaks with the highest VIP scores were m/z 1850.87 and m/z 2164.54. Five peaks were close to the score of 2 (m/z 2307.33, m/z 1740.09, m/z 2068.44, m/z 2200.64 and m/z 2301.44).

### 3.5 CRC EVs Induce Invasion of CRC Cells in Lung Organotypic Model

**FIGURE 5:**
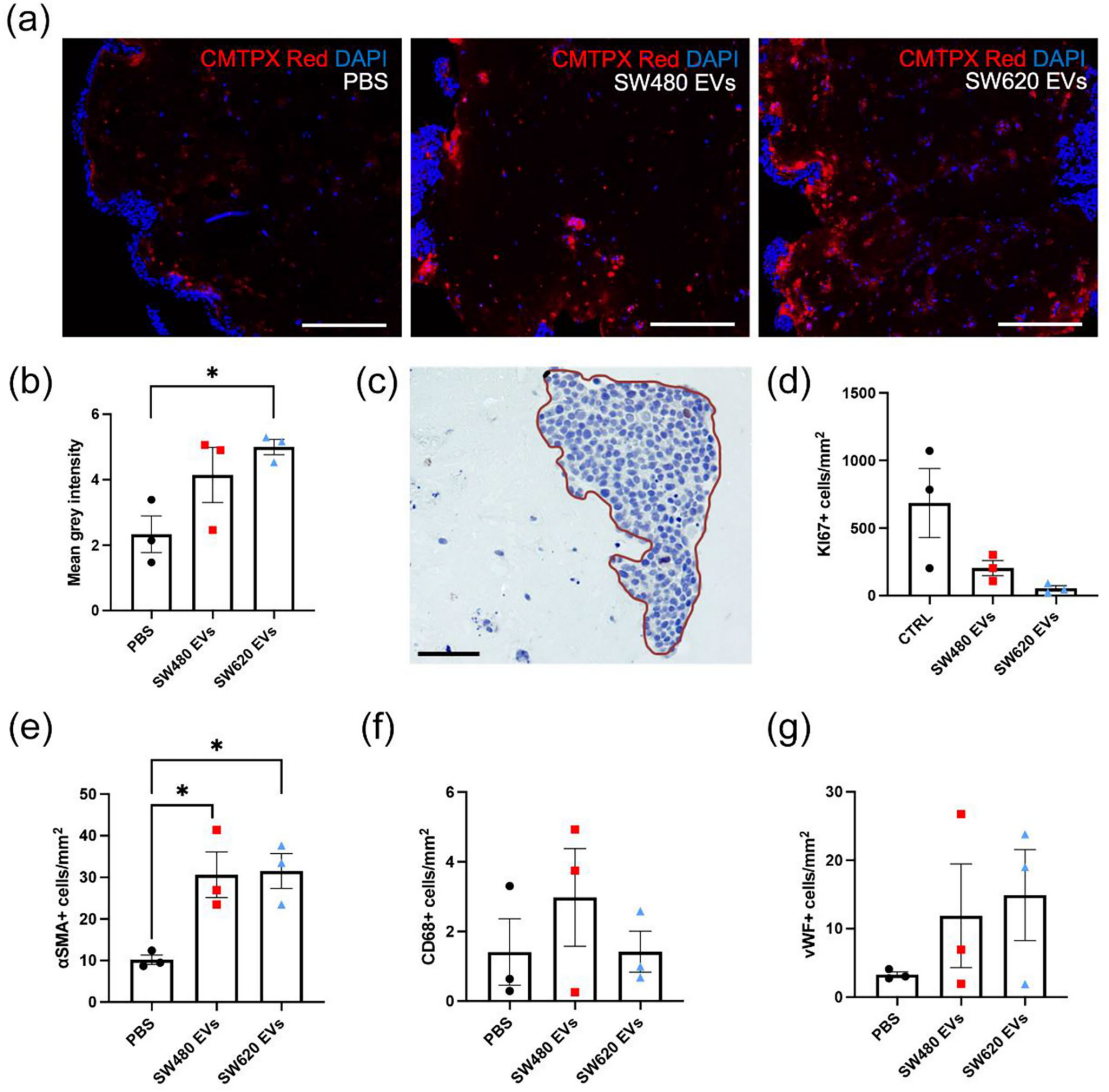
CRC EVs conditioning on CRC invasion in lung organotypic model. (a) Representative images of the lung stroma 3D model after the CRC EVs conditioning and the SW620 cells invasion (red cells). (b) Mean grey intensity of celltracker CMTPX Red staining. Individual values + mean (*n* = 3); ANOVA, * *p* < 0.05. Cell count normalised to mm2. (c) Representative image of SW620 cell cluster ROI for Ki67 expression. (d) Ki67 expression in the SW620 clusters. Individual values + mean (*n* = 3). ANOVA. (e) αSMA expression. Individual values + mean (*n* = 3); ANOVA * *p* < 0.05. (f) CD68 expression. Individual values + median (*n* = 3). KW. (g) vWF expression. Individual values + mean (*n* = 3). ANOVA.

SW620 cells labelled with CellTracker were added onto the lung 3D model after 7 days of EV conditioning and allowed to invade for 4 days before sample processing. Clusters of SW620 cells were found to adhere to the border of the gel in all the conditions, but the number of labelled cells was considerably higher upon EV treatment with mean fluorescence intensity increasing when gels were treated with the latter being statistically significant (*p* < 0.05) (Figure 5A, B).

Expression of α‐SMA, vWF, CD68 and Ki67 were investigated again after the invasion of SW620 cells. Expression of Ki67 was evaluated only in the ROI defined by the SW620 cell clusters (Figure 5C). A small amount of proliferating SW620 remained after the treatment with SW480 (*p* = 0.1401) and SW620 EVs (*p* = 0.0577) compared to PBS treatment (Figure 5C, D). Both CRC EVs induced a statistically significant increase in α‐SMA expression (*p* < 0.05) (Figure 5E). Variable expression of CD68 was observed, especially with SW480 EVs (Figure 5F). An increase in the expression of vWF was observed but failed to reach significance (*p* = 0.3927) (Figure 5G).

## 4 Discussion

This study demonstrates that CRC‐derived EVs strongly influence invasion, ECM remodelling, and stromal activation in 3D organotypic models of cancer progression. By modelling both primary and metastatic conditions, we provide new insight into how EVs modulate tumour—stroma interactions and promote invasion within a physiologically relevant environment, and by adopting two model systems (organotypic invasion and conditioned lung microenvironment), we have been able to separately assess the direct effect of CRC EVs on invasive behaviour of CRC cells and the impact of CRC EVs on generating a permissive microenvironment that subsequently supports CRC invasion. There is growing mechanistic understanding of the individual cellular interactions through which EVs shape the CRC metastatic niche (Wu et al. 2024), but few studies have visualised this in organotypic models to unpick the dynamic, complex TME. Animal studies have suggested that CRC EVs condition the liver through a mechanism involving miRNA driving an inflammatory PMN (Shao et al. 2018), however our model focussed on lung metastasis did not see inflammatory involvement suggesting that invasive patterns and inflammatory conditioning are independent.

In the invasion model, exposure to a high concentration of CRC‐derived EVs (50 µg/ml) significantly enhanced invasion, particularly when derived from metastatic SW620 cells. This agrees with previous evidence that metastatic EVs carry a pro‐invasive cargo that promotes ECM degradation and cellular motility (Costa‐Silva et al. 2015; Giusti et al. 2013; Hoshino et al. 2015; Lafitte et al. 2019; Liu et al. 2020; Luoto et al. 2023; Payton et al. 2021; Raimondo et al. 2015; Rigogliuso et al. 2010; Schillaci et al. 2017a; Schillaci et al. 2017b; Si et al. 2024, Sun et al. 2018, Xu et al. 2024). The greater effect of SW620 EVs compared with SW480 EVs suggests that the stage of the donor tumour determines EV composition and functional impact, reinforcing the concept that tumour progression is accompanied by a qualitative evolution of EV‐mediated signalling (Becker et al. 2016). This also supports the hypothesis of a feedback mechanism, where metastatic EVs could accelerate primary tumour progression or relapse through systemic communication.

In the lung metastasis organotypic model, modelled by increasing cellular complexity through the addition of macrophage‐like and endothelial‐like components, invasion was still promoted by CRC EV treatment but not as significantly as the simpler invasion model. This potentially points to the complex cellular cross‐talk in the metastatic microenvironment. CRC EVs increased gel transparency, implying changes in collagen network density. Although histological analysis did not show overt structural disruption, subtle ECM remodelling or softening is likely, as suggested by the formation of cellular aggregates. Further biomechanical assays, such as atomic force microscopy, will be required to quantify EV‐induced changes in tissue stiffness. Preconditioning of the lung microenvironment with CRC EVs also enhanced the adhesion and localization of SW620 cells at the gel borders, consistent with the formation of a pro‐metastatic niche (Peinado et al. 2017; Ramamoorthy et al. 2019).

At the molecular level, metastatic EVs increased cadherin 2 and delta catenin 1 expression, supporting their role in promoting epithelial—mesenchymal transition (EMT) and invasive phenotypes. Interestingly, cadherin 1 levels were also elevated after EV treatment, contrasting with its expected downregulation during EMT (Thiery et al. 2009). This could be explained by the presence of cadherin 1 on the EV surface (Tang et al. 2018), highlighting the challenge of distinguishing between cellular and vesicular protein sources in immunofluorescence‐based 3D systems. Cadherin 1 and delta catenin 1 are both known to be expressed by EVs secreted from these cell lines, and are upregulated in SW620 cell microvesicles and small exosomes. Cadherin 2, however, is not (Suwakulsiri et al. 2024). Such findings underline the importance of complementary biochemical and imaging methods to confirm the cellular origin of specific proteins, but also point to mediators that could be involved in the distribution and uptake of EVs within the TME, a process also known to involve specific integrins also expressed on EVs from these cells (Hoshino et al. 2015, Suwakulsiri et al. 2024).

Among ECM components, only collagen type XI (Col XI) showed significant modulation, with reduced expression following SW620 EV treatment. This selective alteration may indicate increased collagen turnover or degradation, as cancer EVs often transport matrix metalloproteinases (MMPs) or regulate their activity (Deryugina and Quigley 2015; Shimoda 2019). Integration of protein profiling with MALDI‐MS analyses further revealed a clear separation between SW620 EV‐treated samples and controls, confirming that metastatic EVs induce distinct ECM remodelling patterns. Although peptide identification requires further MS/MS validation, this approach demonstrates the potential of MALDI‐based proteomics for studying ECM alterations in 3D models.

CRC EVs also activated stromal components, as indicated by increased α‐smooth muscle actin (α‐SMA) expression, suggesting fibroblast activation and the emergence of cancer‐associated fibroblasts (CAFs). Such activation appears to occur synergistically with cancer invasion, consistent with previous reports of EV‐mediated fibroblast reprogramming (Costa‐Silva et al. 2015). Similarly, von Willebrand factor (vWF) expression increased near invasive areas, suggesting endothelial recruitment rather than generalised activation. While macrophage (CD68) density remained unchanged, this does not exclude phenotypic polarisation towards pro‐tumorigenic states (Mantovani et al. 2022). Notably, Ki67 staining revealed reduced proliferation in EV‐treated SW620 cells, indicating a possible switch from a proliferative behaviour in favour of an invasive phenotype.

Collectively, these findings highlight multiple roles of CRC‐derived EVs in promoting invasion, ECM remodelling, and stromal activation within 3D microenvironments. The differential effects of SW480 and SW620 EVs emphasize the importance of tumour stage‐specific vesicle signatures and their contribution to local and systemic disease progression. Further studies expanding donor EVs beyond this cell line model and into primary patient‐derived sources, and integrating mechanical profiling, proteomics and single‐cell transcriptomics will be essential to dissect the molecular mechanisms underlying EV‐mediated remodelling and to identify potential therapeutic targets that disrupt this pro‐metastatic communication network.

## Author Contributions

**Sonia Guarnerio**: data curation (lead), formal analysis (lead), investigation (lead), methodology (lead), visualisation (lead), writing – draft preparation (lead), writing – review and editing (joint). **Laura Cole**: conceptualisation (supporting), data curation (supporting), funding acquisition (supporting), project administration (supporting), supervision (supporting), visualization (supporting), writing – review and editing (joint). **Rawan Maani**: data curation (supporting), investigation (supporting). **Rob Tempest**: data curation (supporting), investigation (supporting). **Alex Mirnezami**: conceptualisation (supporting), funding acquisition (supporting), methodology (supporting). **Paul Hughes**: methodology (supporting), data curation (supporting), formal analysis (supporting), writing – review and editing (supporting). **Stuart Hunt**: conceptualization (supporting), funding acquisition (supporting), supervision (supporting), visualization (supporting), writing – review and editing (joint). **Christine Le Maitre**: conceptualisation (supporting), funding acquisition (supporting), methodology (supporting), project administration (supporting), supervision (supporting), visualization (supporting), writing – review and editing (joint). **Keith Chapple**: Resources (joint), writing – review and editing (joint). **Nick Peake**: conceptualisation (lead), funding acquisition (lead), methodology (supporting), project administration (lead), resources (joint), supervision (lead), visualization (supporting), writing – original draft preparation (supporting), writing – review and editing (joint).

## Conflicts of Interest

The authors declare no conflicts of interest.

## Supporting information




**Supporting information**: jex270187‐sup‐0001‐FigureS1‐S8.pdf

## Data Availability

The data that support the findings of this study are available from the corresponding author upon reasonable request (the institutional repository holds all data and facilitates data sharing as detailed in methods).
