# Supplementary material for: Extracellular Vesicles Drive Invasive Characteristics and Matrix Remodelling in Organotypic Models of Colorectal Cancer Progression
Source: J Extracell Biol. 2026 Sep 17;5(9):e70187. doi: 10.1002/jex2.70187 (PMC13586556; doi:10.1002/jex2.70187)
Supplement: Supplementary file 1 [file JEX2-5-e70187-s001.pdf]

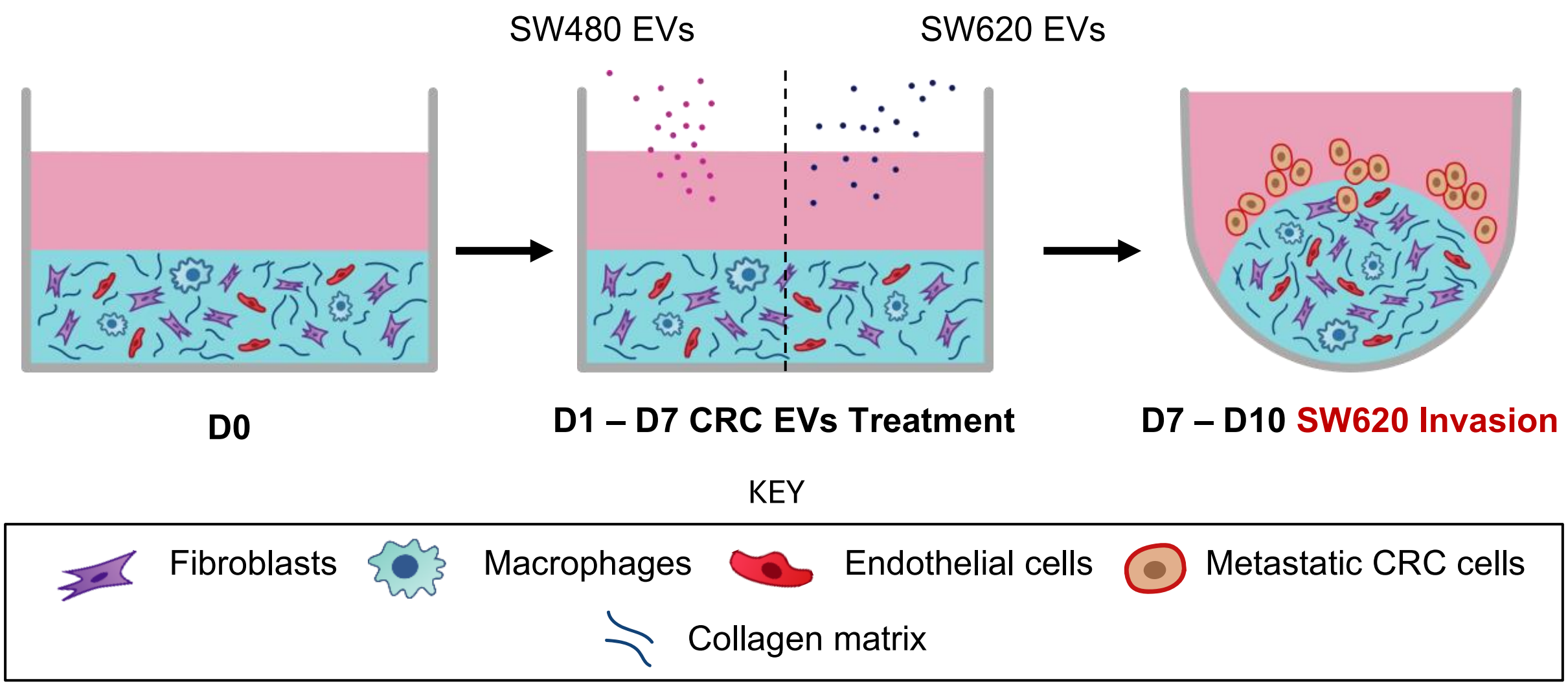

Supplementary figure 1: graphical representation of lung stromal model, illustrating the conditioning of lung stromal cells by CRC EVs, and subsequent assessment of invasion by the CRC cell line SW620.

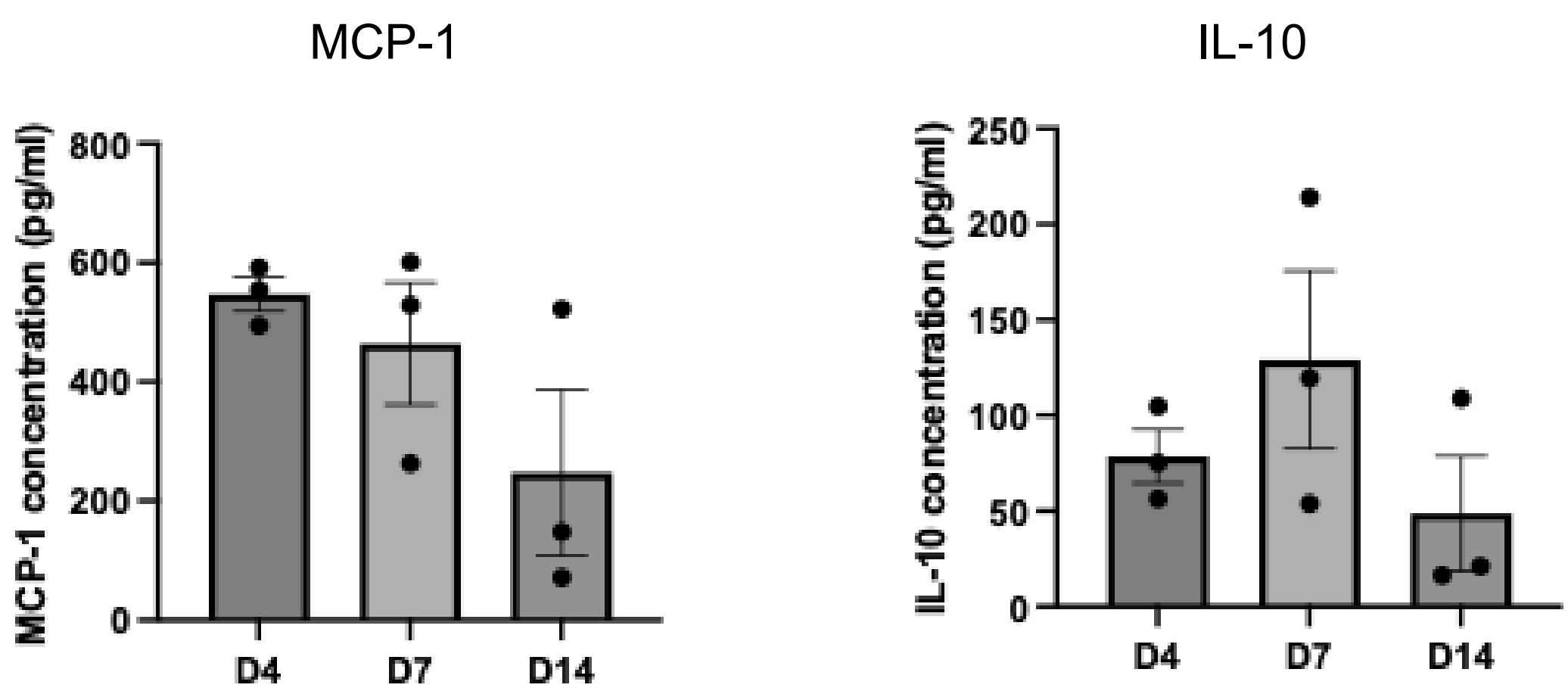

Supplementary figure 2: Release of MCP-1 and IL-10 from lung stromal 3D models.

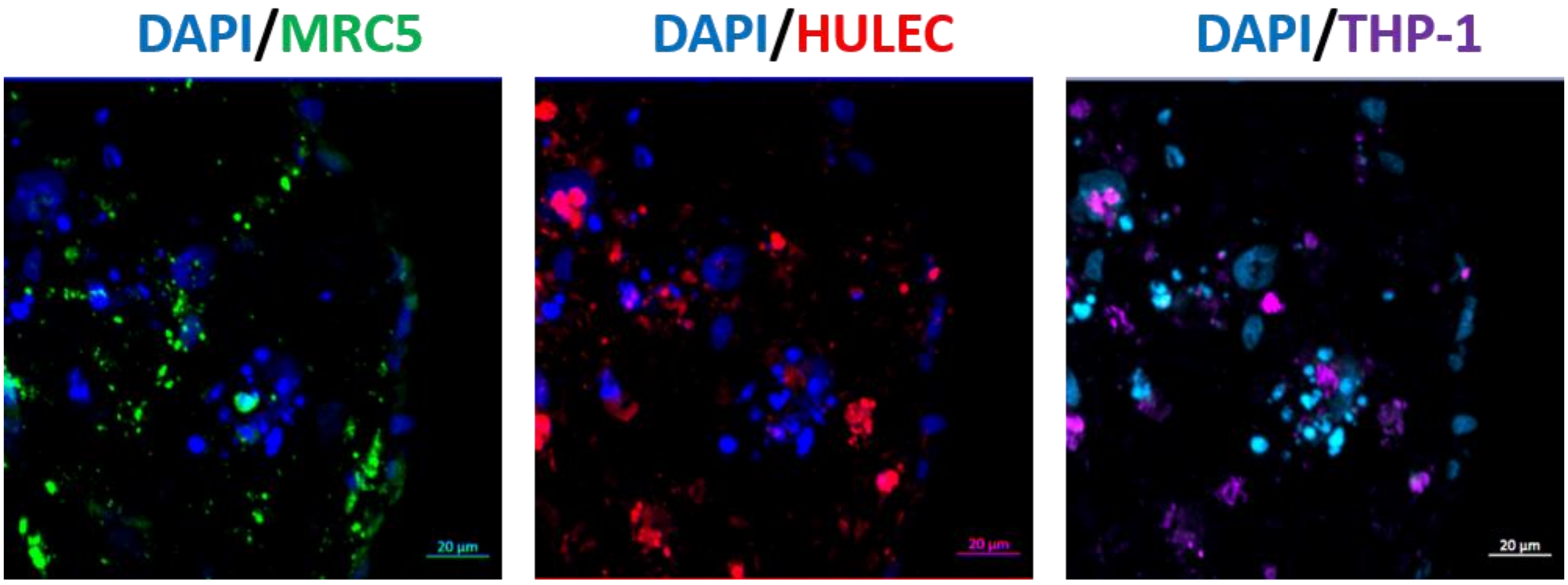

Supplementary figure 3: Individual DAPI counter-stained images of celltracker-labelled cells in the lung stromal model after 7 days of culture.

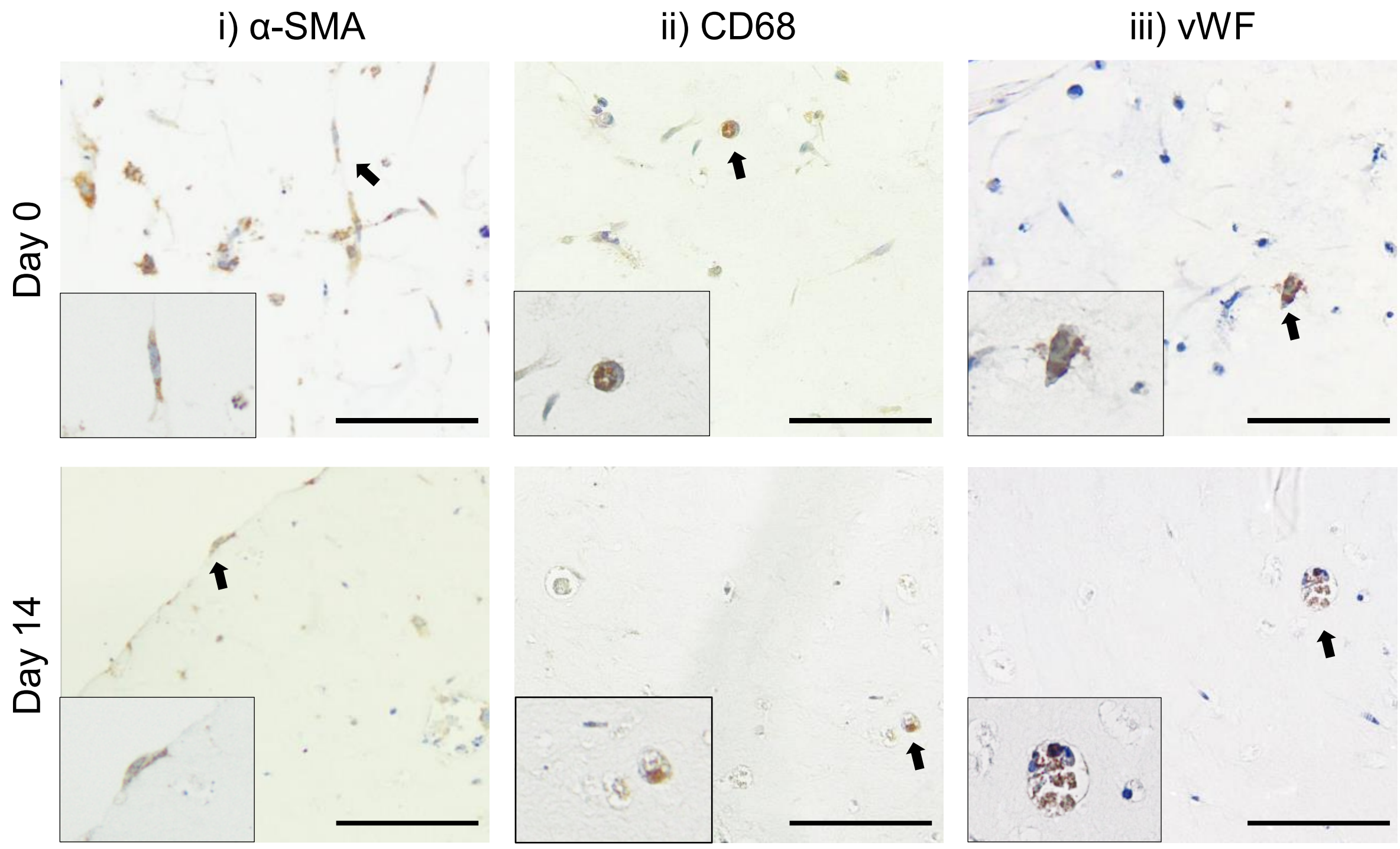

Supplementary figure 4: Expression of key cellular markers in lung stromal 3D model.

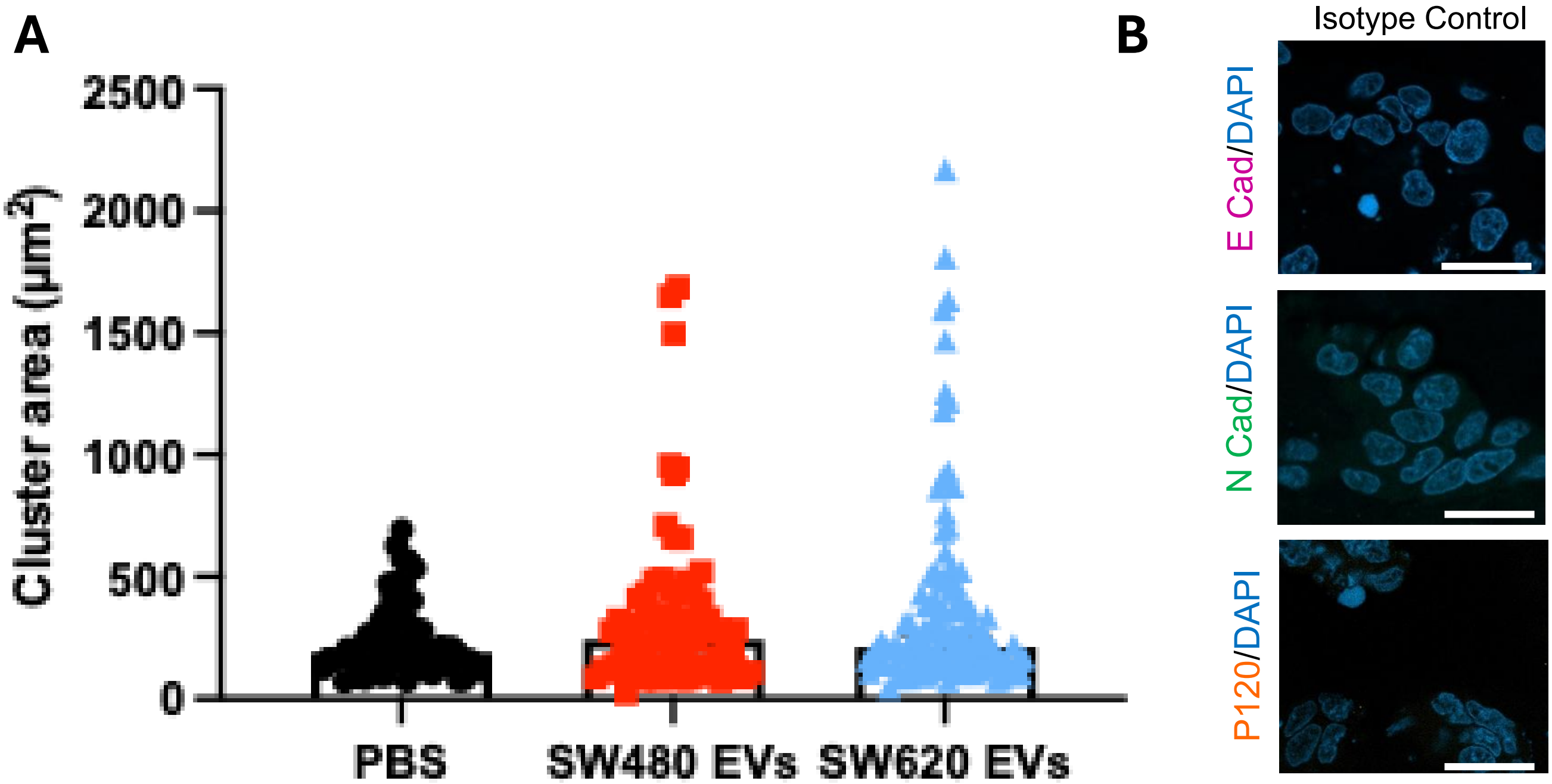

Supplementary figure 5: The cluster area size after treatment with EVs trended towards being higher after treatment with both SW480 and SW620 EVs, but did not reach statistical significance (A). Isotype controls for staining with anti cadherin and p120 antibodies showed low levels of background (B).

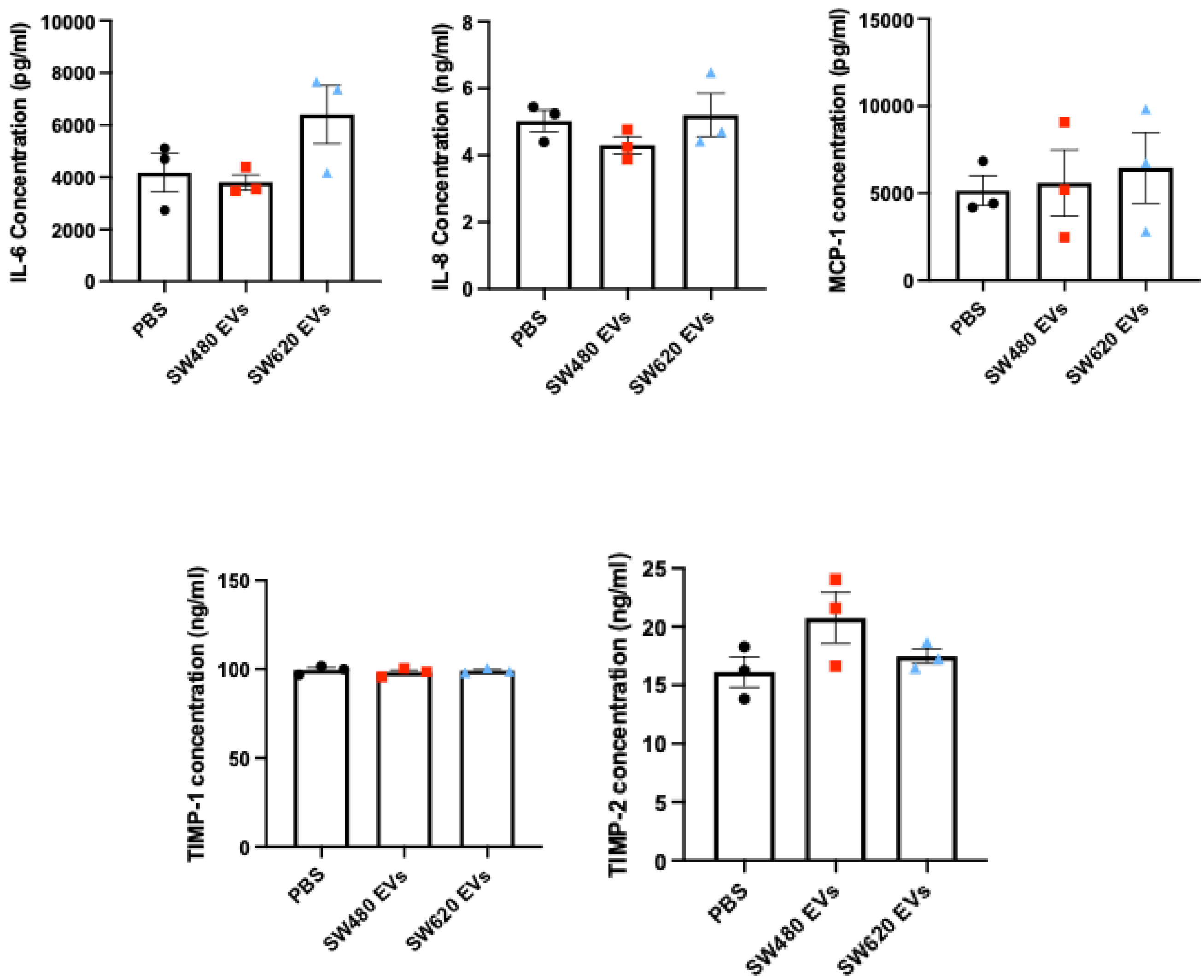

Supplementary figure 6: Release of inflammatory cytokines and the ECM regulatory proteins TIMP-1 and TIMP-2 from models after treatment with SW480 and SW620 EVs.

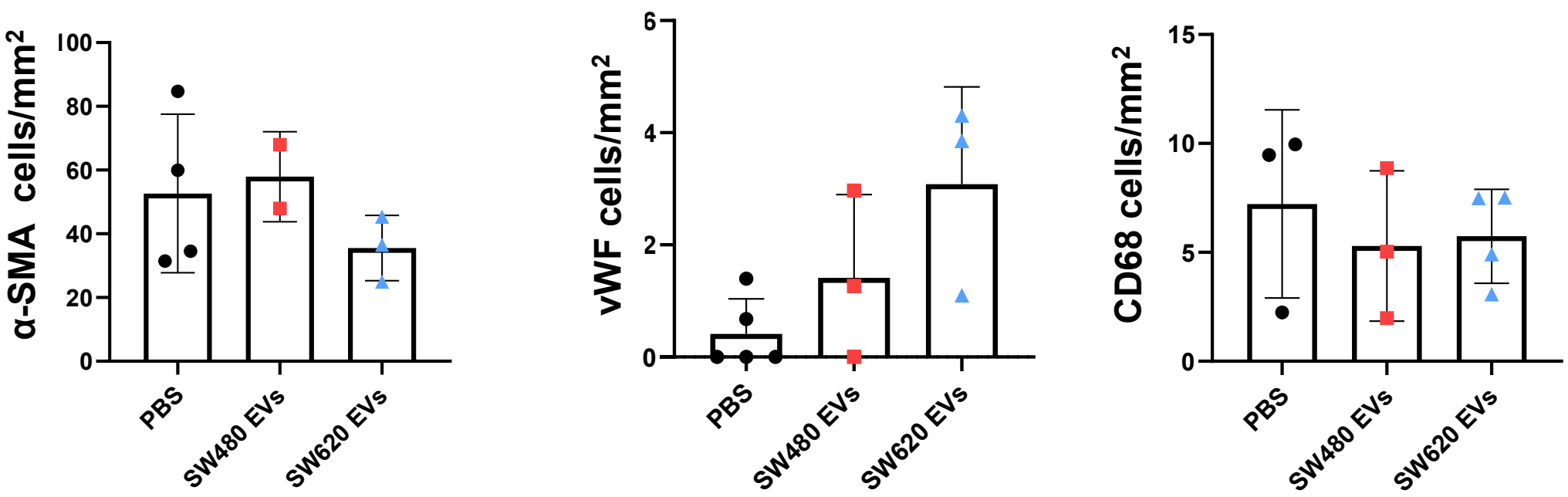

Supplementary figure 7: Quantitation of expression of key cellular marker proteins in lung stromal model.

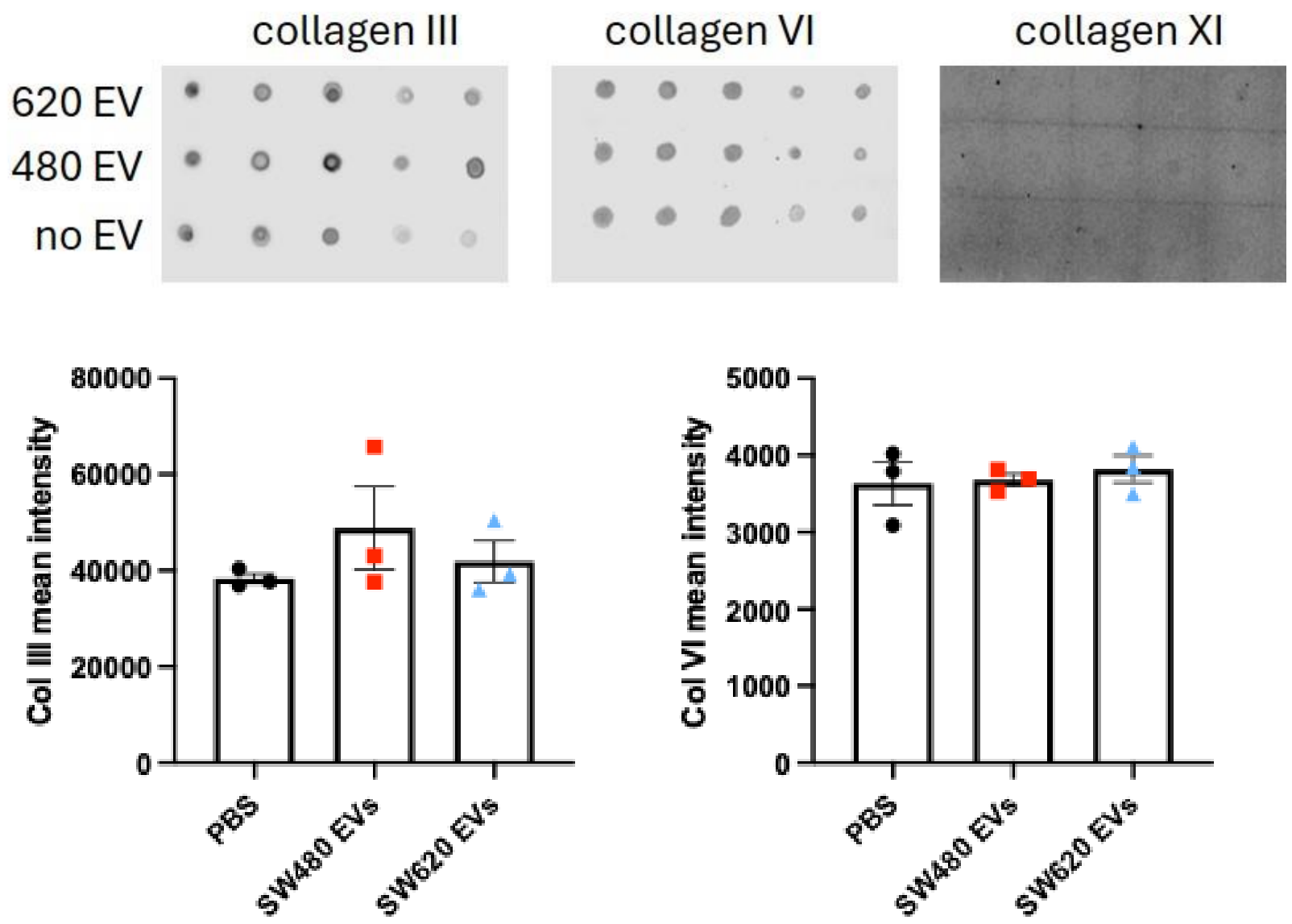

Supplementary figure 8: Quantitation of expression of key collagens by dot blot in lung stromal model.
